# Early Functional Recovery Is Improved in Patients Treated With Bioinductive Collagen Implant Augmentation Compared With Standard Arthroscopic Repair of High-grade Partial-Thickness Rotator Cuff Tears: A Prospective Randomized Trial

**DOI:** 10.1177/23259671261418675

**Published:** 2026-03-04

**Authors:** Allan Wang, William Breidahl, Eugene T. Ek, Travis Falconer, Peter D’Alessandro, Jay R. Ebert

**Affiliations:** * Department of Orthopaedic Surgery, St John of God Subiaco Hospital, Perth, Western Australia, Australia; † Department of Orthopaedics, University of Western Australia, Perth, Western Australia, Australia; ‡ Perth Radiological Clinic, Perth, Western Australia, Australia; § Melbourne Orthopaedic Group, Melbourne, Victoria, Australia; ‖ Department of Surgery, Monash Medical Center, Monash University, Melbourne, Victoria, Australia; ¶ Perth Orthopaedic & Sports Medicine Centre, Perth, Western Australia, Australia; # Orthopaedic Research Foundation of Western Australia, Perth, Western Australia, Australia; ** Fiona Stanley and Fremantle Hospitals Group, South Metropolitan Health Service, Perth, Western Australia, Australia; †† School of Surgery, University of Western Australia, Perth, Western Australia, Australia; ‡‡ School of Human Sciences (Exercise and Sport Science), University of Western Australia, Perth, Western Australia, Australia; §§ HFRC Rehabilitation Clinic, Perth, Western Australia, Australia; Investigation performed at the St John of God Hospital, Perth, Western Australia, Australia

**Keywords:** rotator cuff partial-thickness tear, bioinductive collagen implant, clinical outcomes, magnetic resonance imaging

## Abstract

**Background:**

Arthroscopic surgical takedown and repair of symptomatic partial-thickness rotator cuff tears are commonly undertaken. An alternative approach is the use of a bioinductive collagen implant to augment the rotator cuff tear.

**Purpose:**

To investigate early function and rotator cuff tendon integrity in patients undergoing arthroscopic bioinductive collagen implant augmentation (REG group) versus rotator cuff takedown and repair (RCR group) for high-grade partial-thickness rotator cuff tears.

**Study Design:**

Randomized controlled clinical trial; Level of evidence, 2.

**Methods:**

Patients 35 to 75 years of age with symptoms >3 months and unresponsive to nonoperative treatment, with high-grade partial-thickness rotator cuff tears confirmed on 3-T magnetic resonance imaging (MRI), were randomly allocated to RCR or REG groups. Exclusion criteria included previous ipsilateral shoulder surgery, multitendon tears/pathology, and concomitant surgery including labral repair, long head of biceps tenodesis, or chondroplasty/microfracture. Patients were assessed preoperatively and at 6 weeks as well as 3, 6, and 12 months postoperatively. The primary study outcome was the Western Ontario Rotator Cuff Index (WORC) at 3 months after surgery. Secondary outcomes included the American Shoulder and Elbow Surgeons (ASES) and Constant scores, and the time taken to return to work and activities of daily living (ADLs). MRI-based rotator cuff repair integrity was assessed using the Sugaya grading system.

**Results:**

This study recruited 41 patients (REG n = 21; RCR n = 20). No group differences (*P* > .05) were observed in mean age (REG 57.1 years; RCR 57.8 years), preoperative duration of symptoms, and previous nonoperative treatments. The REG group reported superior outcomes for the WORC at 6 weeks (*P* = .001) and 3 months (*P* = .026) as well as the ASES at 6 weeks (*P* < .001) and WORC Work, Sport, and Emotions domains at 6 weeks and 3 months (*P* < .05). There were no group-based differences (*P* > .05) in patient-reported outcomes at 6 or 12 months after surgery. The REG group was faster (*P* < .05) to permanently remove the sling, drive a motor vehicle, and return to office duties and moderate-intensity chores. No MRI-based differences were observed, with 19 (90%) and 17 (85%) REG and RCR patients, respectively, graded Sugaya 1 to 2 at 12 months.

**Conclusion:**

For symptomatic high-grade partial-thickness rotator cuff tears, bioinductive collagen implant augmentation versus standard rotator cuff repair demonstrated improved early function with equivalent MRI-based healing rates.

**Registration:**

Australian New Zealand Clinical Trials Register (ACTRN12620000926932p).

Partial-thickness rotator cuff tears are prevalent in middle-aged individuals and may be asymptomatic.^13,22^ When patients present with symptomatic partial-thickness rotator cuff tears and subacromial impingement syndrome, nonoperative treatment can be successful. However, Lo et al^15^ reported on factors that were predictive of failure of nonoperative treatment, including involvement of the dominant shoulder, traumatic onset of symptoms, and a high-grade partial-thickness rotator cuff tear, which has been defined as involving >50% of tendon thickness on diagnostic imaging.^1,15^ A high-grade partial-thickness rotator cuff tear may remain symptomatic due to the increase in peak intratendinous strain in the adjacent intact cuff tendon.^18,20^

For symptomatic partial-thickness rotator cuff tears that are unresponsive to nonoperative treatments, the standard surgical approach is completion of the rotator cuff tear, debridement of the tendon tissue, and repair, followed by sling immobilization for 4 to 6 weeks.^17,23^ An in situ repair is a further well-detailed surgical approach to treating high-grade supraspinatus tendon tears but may be associated with high rates of shoulder stiffness and inferior functional scores.^11,23^ Takedown of partial-thickness rotator cuff tear sacrifices the remaining rotator cuff inserting onto the tuberosity, including the fibrocartilage enthesis, which contributes mechanical support for the cuff tendon insertion to the tuberosity. The rotator cuff tendon retear rate after takedown and repair of a high-grade partial-thickness tear has been reported as high as 11.9% to 13.5%.^5,12^ Repair of the partial-thickness rotator cuff tear by advancement to the tuberosity may also increase the risk for postoperative stiffness.^8^ These concerns have led to the development of other approaches to the surgical treatment of partial-thickness rotator cuff tears.

A bioinductive collagen implant, arthroscopically applied to the bursal surface of the partial-thickness rotator cuff tear in the subacromial space, has shown promising early outcomes and low complication rates in case series, registry studies, and reviews.^2,3,16,21,26^ The potential benefits of collagen implant augmentation over standard rotator cuff takedown and repair may be seen in the early stages of recovery, including earlier return to activities of daily living (ADLs), work, and recreational activities.^3,16^ McIntyre et al^16^ reported results of the REBUILD registry for bioinductive collagen implant treatment of partial-thickness rotator cuff tears and noted superior 3-month postoperative American Shoulder and Elbow Surgeons (ASES) scores compared with previously published studies of rotator cuff takedown and repair. Bushnell et al^3^ also reported outcomes of bioinductive collagen implant treatment for partial-thickness rotator cuff tears from the REBUILD registry, including Western Ontario Rotator Cuff Index (WORC) and ASES scores and timelines to return to ADLs. Those investigators further noted that a direct comparative study of functional recovery versus standard rotator cuff takedown and repair was yet to be undertaken to demonstrate the potential advantages of bioinductive collagen implant augmentation for high-grade partial-thickness rotator cuff tears. Data from a comparative study will further permit the appropriate counselling of patients regarding surgical treatment options and expected postoperative timelines for the safe return to various work, recreational, and sporting activities.

The aims of the current prospective, randomized trial were to investigate early symptoms and timelines for functional recovery in patients undergoing bioinductive collagen implant augmentation versus standard arthroscopic rotator cuff takedown and repair for the treatment of symptomatic, high-grade, partial-thickness supraspinatus tears. The primary aim was to investigate WORC score at 3 months after surgery, with the primary hypothesis that patients undergoing bioinductive collagen implant augmentation, compared with standard rotator cuff repair, would demonstrate a superior 3-month WORC. Secondary aims included the evaluation of differences between bioinductive collagen implant augmentation and rotator cuff repair in patient-reported outcome measures (PROMs); the timing of return to various ADLs, work, recreational, and sporting activities; and tendon healing up until 12 months after surgery. The secondary hypotheses were that patients undergoing bioinductive collagen implant augmentation would demonstrate superiority in other PROMs and an earlier return to various ADLs, work, recreational, and sporting activities, with an equivalent rate of tendon healing at 12 months after surgery.

## Methods

Ethical approval for this prospective randomized trial was provided by the Human Research Ethics Committee (HREC) of St John of God Health Care (ID 1687) and Ramsay Health Care (ID 2020-011). The trial was prospectively registered with the Australian New Zealand Clinical Trials Registry (ACTRN12620000926932p). Patients enrolled in this trial were randomly allocated to either (1) arthroscopic decompression and takedown of the partial-thickness rotator cuff tendon tear with a double-row surgical repair (RCR group) or (2) arthroscopic decompression with augmentation of the partial-thickness rotator cuff tear with a bioinductive collagen implant (REG group). An online platform (Sealed Envelope) was used to undertake prospective randomization, controlled by an independent researcher.

### Patients

A consecutive series of patients presenting with symptomatic, partial-thickness supraspinatus tears, who fulfilled the inclusion and exclusion criteria outlined below and who consented to enter the trial, were enrolled into the study by 1 of 4 orthopaedic surgeons. To be included in the study, patients were between the ages of 35 and 75 years, had a history of symptoms >3 months, and presented with a high-grade (≥50%) partial-thickness supraspinatus tear as demonstrated on a 3-T magnetic resonance imaging (MRI) scan^1,15^ that, in combination with patient-reported symptoms and clinical examination by the orthopaedic surgeon, confirmed the rotator cuff tear as the primary pathology driving pain, symptoms, and functional disability. Although the specific duration, frequency, and content were not assessed, all patients had undergone nonoperative treatment including a trial of physical therapy without resolution of shoulder symptoms. Furthermore, all patients had undergone oral anti-inflammatory medication and/or corticosteroid subacromial injection at least 3 months before the scheduled surgery. Exclusion criteria included previous surgery on the ipsilateral study limb, multitendon pathology including partial-thickness tears to subscapularis or infraspinatus tendons, and the need for concomitant surgery including labral repair, long head of biceps tenodesis, or chondroplasty/microfracture. Patients with long head of biceps pathology noted preoperatively on clinical examination and MRI evaluation who had consented to biceps tenotomy were not excluded from trial participation. Patients were advised of the treatment options required to address their long head of biceps pathology and that long-term clinical outcomes of biceps tenodesis or tenotomy were equivalent. However, patients were also informed that the choice of tenodesis excluded them from trial participation, due to the additional requirement to protect the repair. In addition, those patients with concomitant pathology including any glenohumeral arthritis noted on MRI, nerve compression, adhesive capsulitis, shoulder instability, previous fracture, or infection affecting the shoulder joint were excluded. Furthermore, lactating or pregnant patients, professional athletes, patients presenting under a workers’ compensation scheme, current smokers, or patients with a known substance abuse concern or mental illness were excluded.

Patients who fulfilled the inclusion and exclusion criteria were informed by the surgeon initially of the trial protocol and requirements for participation in the trial and were subsequently provided with written information approved by the relevant HREC. Patients were provided the opportunity to discuss the written information and trial participation with family or other medical professionals. Prospective participants were then interviewed by an independent research associate, and details and requirements associated with the trial were further discussed. If the patient wished to proceed and participate in the trial, the patient was then consented and randomized by the online platform (Sealed Envelope) before preoperative assessment and data collection. The patient was then subsequently reviewed by the surgeon and consented for the allocated surgical procedure. The treating surgeon, trial coordinator, and patient were not blinded to treatment group allocation.

### Surgical Procedures and Rehabilitation

The surgical procedures were performed with patients in either the beach-chair or lateral decubitus position, depending on surgeon preference. A general anesthetic with interscalene brachial plexus blockade was used in all cases. Prophylactic antibiotics (cefazolin 1 g IV) were administered. Arthroscopic inspection of the supraspinatus tendon from the glenohumeral joint and subacromial viewing portals confirmed that the partial-thickness rotator cuff tear was high-grade and predominantly either articular sided, bursal sided, or interstitial. The arthroscopic finding was correlated with the MRI scan. No patients were excluded from trial participation at the time of surgery on the basis of unexpected arthroscopic findings, including a full-thickness tendon tear, arthritis, or biceps or other rotator cuff tendon pathology not identified at the time of preoperative clinical and MRI-based evaluation.

A 16-gauge spinal needle was passed percutaneously through the articular side of the partial-thickness rotator cuff tear and into the glenohumeral joint under direct arthroscopic vision. A PDS suture was placed through the needle to mark the partial-thickness rotator cuff tear site. In the subacromial space, a bursectomy was performed and the partial-thickness rotator cuff tear was clearly identified visually and by probing. An arthroscopic acromioplasty was performed in all cases. Concomitant acromioplasty at the time of rotator cuff repair is controversial. Although some studies have shown no difference in long-term outcomes of rotator cuff repair with and without acromioplasty,^27,28^ a recent systematic review by Yang et al^29^ reported that concomitant acromioplasty was associated with superior PROMs and a lower reoperation rate.

In patients randomized to the RCR group, the partial-thickness rotator cuff tear was fully released from the greater tuberosity. The bony footprint was debrided of residual tendon and lightly decorticated with a shaver. The tendon free edge was debrided with a duck bill punch or shaver to thicker and healthy tendon tissue. A 2.4-mm double-loaded suture anchor was placed 2 to 3 mm lateral to the articular margin, and sutures were passed through the free edge of supraspinatus using a suture passer. Sliding medial row knots were tied to appose the supraspinatus tendon to the medial footprint. The suture ends were secured with 1 to 2 lateral row anchors, completing a double-row cuff tendon repair. For the RCR group, patients were generally discharged from hospital on the day after surgery, with a sling worn 24 hours per day for the first week, permitting no shoulder movement. At the beginning of week 2, a rehabilitation program was commenced with passive range of motion (ROM) exercises initiated in forward flexion and external rotation and progressed as symptoms allowed. At 5 weeks after surgery, patients were allowed to discard the sling and were permitted active-assisted and active elevation and rotation exercises, aiming for full active ROM at 10 weeks. Graded resistance and strengthening exercises with terminal range stretches were then undertaken from 8 to 10 weeks, inclusive of internal rotation. At 16 weeks, patients were allowed to return to full and unrestricted activity as tolerated including moderate to heavy manual tasks, although no further specific criteria were used to permit the release to unrestricted activities.

In patients randomized to the REG group, the technique used for arthroscopic placement of the bioinductive collagen implant (Regeneten; Smith & Nephew) on the bursal surface of the partial-thickness supraspinatus tendon tear closely followed previous published techniques.^3,16^ The proprietary, single-use, disposable implantation devices were used. As with the RCR group, the partial-thickness supraspinatus tendon tear was positively identified from glenohumeral and subacromial viewing portals. After acromioplasty and bursal debridement, conservative cuff debridement of frayed and unstable tendon fibers was performed on the articular or bursal aspect. The medium-sized (20 × 25 mm) bioinductive collagen implant was used in all cases. Poly-l-lactic acid tendon anchors were used medially, and 2 PEEK bone anchors were used laterally. For the REG group, patients were discharged from hospital on the day after surgery, with a sling worn 24 hours per day for the first week. At the beginning of week 2, the sling could be discarded as symptoms allowed. Patients then followed a progressive rehabilitation program of ROM and resistive exercises as previously published.^3,16^ No internal rotation of the hand behind the back was permitted. At 4 weeks, active ROM exercises were permitted, and at 6 weeks strengthening exercises were introduced. At 12 weeks, full and unrestricted activity was generally permitted as tolerated.

If clinically indicated, concomitant surgery was undertaken including long head of biceps tenotomy (RCR group = 4; REG group = 5) and acromioclavicular joint debridement (RCR group = 4; REG group = 2). No concomitant long head of biceps tenodeses or labral repairs were performed in either group. Concomitant surgery, when performed, did not change the sling mobilization or postoperative rehabilitation program in either treatment group.

### Clinical Evaluation

Preoperatively, a range of variables relating to patient demographics and injury history were collected, including age, sex, height, weight, body mass index, operative side, dominant side (preferred throwing arm), duration of symptoms, injury mechanism (if known), previous nonoperative treatments, and/or analgesic exposure (including physical therapy or other allied health treatments, nonsteroidal anti-inflammatory drugs, and corticosteroid injections).

PROMs were collected before surgery and at 6 weeks after surgery as well as 3, 6, and 12 months after surgery. The WORC total score (primary outcome) was assessed.^14^ The minimally important difference (MID) of the WORC in patients with rotator cuff tears treated surgically has been reported as 282.6 points (on a 0- to 2100-point scale) or 13.5%.^9^ Furthermore, the individual WORC subscales (Symptoms, Sport/Recreation, Work, Lifestyle, and Emotions) were assessed.^14^ Additional PROMs collected included the ASES score to evaluate shoulder pain and function, the Single Assessment Numeric Evaluation (SANE) to determine how the patient rated their shoulder as a percentage of normal (0%-100% scale with 100% being normal), and the Veterans Rand 12-Item Health Survey (VR-12) to evaluate the patient's perception of their general health, producing a Physical Component Score (PCS) and a Mental Component Score (MCS). MIDs after rotator cuff repair of 21.9, 27.3. 2.6, and 1.9 have been reported for the ASES, SANE, VR-12 PCS, and VR-12 MCS, respectively.^9,31^

Preoperatively and at 6 and 12 months after surgery, the Constant score was collected from all patients.^6,7^ The overall Constant score was reported (0-100), as were the individual subdomains including patient-reported pain (0-15 points); the effect of the shoulder condition on occupational, leisure, and other daily activities (0-20 points); active ROM (0-40 points); and maximal pain-free isometric abduction strength in 90° of shoulder abduction in the scapular plane (0-25 points). All clinical assessments were undertaken by an independent assessor (J.E.), a physical therapist with >20 years of experience in orthopaedic research trials, who was not blinded to group allocation.

Finally, a detailed evaluation of specific patient activity and the timing of return to various work, ADLs, recreational, and sporting activities was undertaken on each patient through the postoperative recovery timeline. This included time (days/weeks) before permanently removing the sling, driving a car, returning to work in an office setting, and undertaking light and heavy manual tasks. The tasks investigated in the current study had been reported in previous research.^16^

### Radiological Evaluation

High-resolution MRI was undertaken in patients preoperatively and at 6 and 12 months after surgery using a 3.0-T scanner (Philips 3T Achieva; General Electric 3T Discovery) with 80 mT/m and 50 mT/m gradient power, respectively. Multiple images were obtained in oblique coronal and oblique sagittal planes with both proton density (PD)–weighted and T2-weighted fat-suppressed turbo spin echo sequences. PD-weighted fat-suppressed images were also obtained in the axial plane.

Preoperative MRI was used to identify whether the supraspinatus tendon partial-thickness tear was predominantly articular, bursal, or interstitial in configuration. All partial-thickness supraspinatus tears were high-grade involving ≥50% of the tendon thickness at the greater tuberosity footprint as noted on the T2-weighted coronal oblique images. Further, all postoperative (6- and 12-month) scans were assessed for residual tendinosis or tear severity using the Sugaya classification.^24^ The status of the repair was graded as follows: grade 1 = no tear (sufficient tendon thickness with homogeneously low intensity); grade 2 = sufficient tendon thickness but with partial high intensity; grade 3 = insufficient thickness but no tendon discontinuity; grade 4 = small full-thickness retear (the presence of a minor discontinuity); grade 5 = large full-thickness retear (the presence of a major discontinuity). MRI assessment was performed by a fellowship-trained musculoskeletal radiologist with >20 years of experience who was not informed of the group allocation of participants.

### Data and Statistical Analysis

An a priori power calculation was performed using G-Power based on our designated primary outcome measure: the overall WORC score at 3 months after surgery. A large effect size was anticipated in the WORC given that the primary endpoint was 3 months after surgery and the comparison was between an RCR cohort with an expected 5-week period of sling use versus the REG cohort where the rehabilitation guidance was for 1 week of sling use. For the anticipated large effect (0.9), it was estimated that 40 participants (20 in each group) would be required to reveal differences at the 5% significance level, with 80% power.

The mean ± standard deviation (with range) and number (with percentage) of relevant patient demographic and injury characteristics were calculated for the 2 groups. Independent *t* tests (or chi-square tests for categorical data) were undertaken in pertinent demographics and injury history variables between groups.

The means ± standard deviations of all pre- and postoperative clinical scores were calculated and presented. Repeated-measures analysis of variance (ANOVA) was used to assess differences across the 2 groups over time in clinical scores. Where a significant group or interaction effect was found, post hoc independent *t* tests were used to determine timepoints at which the groups differed. The MRI-based Sugaya gradings (median and range) at 6 and 12 months were presented for the REG and RCR groups, along with the number and percentage of patients graded within each of the Sugaya classifications. The nonparametric Mann-Whitney test was used to estimate group differences.

Where appropriate, statistical analysis was performed using SPSS software (Version 30.0), with significance determined at *P* < .05.

## Results

**Figure 1.**
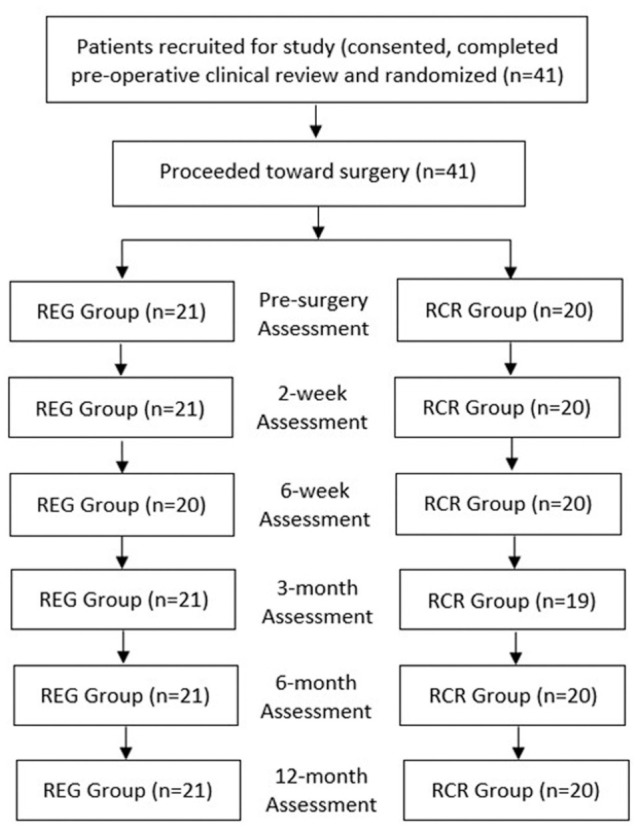
Flowchart demonstrating patient recruitment and patient evaluation over the 12-month follow-up study period, in patients randomized to the 2 groups: standard rotator cuff repair (RCR group) or augmentation of the rotator cuff tear with the bioinductive collagen implant (REG group).

This study recruited 41 participants (RCR group = 20; REG group = 21) (Figure 1). A comparison of baseline measures for recruited participants in the 2 study groups is presented in Table 1, showing no differences between groups in demographic characteristics, injury history variables, or previous nonoperative treatments. Notably, participants had chronic shoulder symptoms with a mean duration of ≥3 years in both groups, despite nonoperative treatments. The distribution of high-grade partial-thickness tears across articular, bursal, and interstitial tendon tear patterns was similar between treatment groups (Table 1). Over the 12-month assessment period, no patient was lost to follow-up. However, 1 patient was unable to attend the 6-week clinical review (REG group), and 1 patient was unable to attend the 3-month clinical review (RCR group). A “last observation carried forward” approach was adopted for these 2 assessment timepoints, and all patients attended all other clinical and radiological reviews.

**Table 1:** Preoperative Demographic Characteristics, Injury History, and Commonly Reported Previous Nonoperative Treatments for Patients Randomized to the 2 Groups*^*a*^*

Variable	REG Group	RCR Group	*P*
n	21	20	—
Age, y	57.1 ± 11.2 (36-73)	57.8 ± 8.1 (37-69)	.816
Weight, kg	78.9 ± 19.2 (47-120)	80.7 ± 18.8 (51-110)	.875
Body mass index, kg/m^2^	26.7 ± 4.7 (17.3-35.4)	27.1 ± 5.2 (17.6-35.5)	.616
Duration of symptoms, mo	35.7 ± 28.0 (4-104)	39.5 ± 54.1 (4-240)	.936
Male, n (%)	11 (52)	11 (55)	.746
Operated limb is dominant side, n (%)	10 (47)	9 (45)	.866
Tear location, n			
Interstitial	9	9	.890
Articular	9	8	.853
Bursal	3	3	.948
Mechanism of injury, n (%)			
Unknown	14 (67)	13 (65)	.910
Activities of daily living	5 (24)	5 (25)	.929
Recreational/sport	2 (9)	2 (10)	.959
Reported previous nonoperative treatments, n (%)			
Physical therapy	14 (67)	16 (80)	.335
Nonsteroidal anti-inflammatory drugs	14 (67)	15 (75)	.558
Injection (corticosteroids)	19 (90)	17 (85)	.592

a Data are expressed as mean ± SD (range) unless otherwise noted. *P* values represent group comparisons. RCR, rotator cuff repair; REG, augmentation of the rotator cuff tear with the bioinductive collagen implant.

### Clinical Scores and Return to Activities

**Figure 2.**
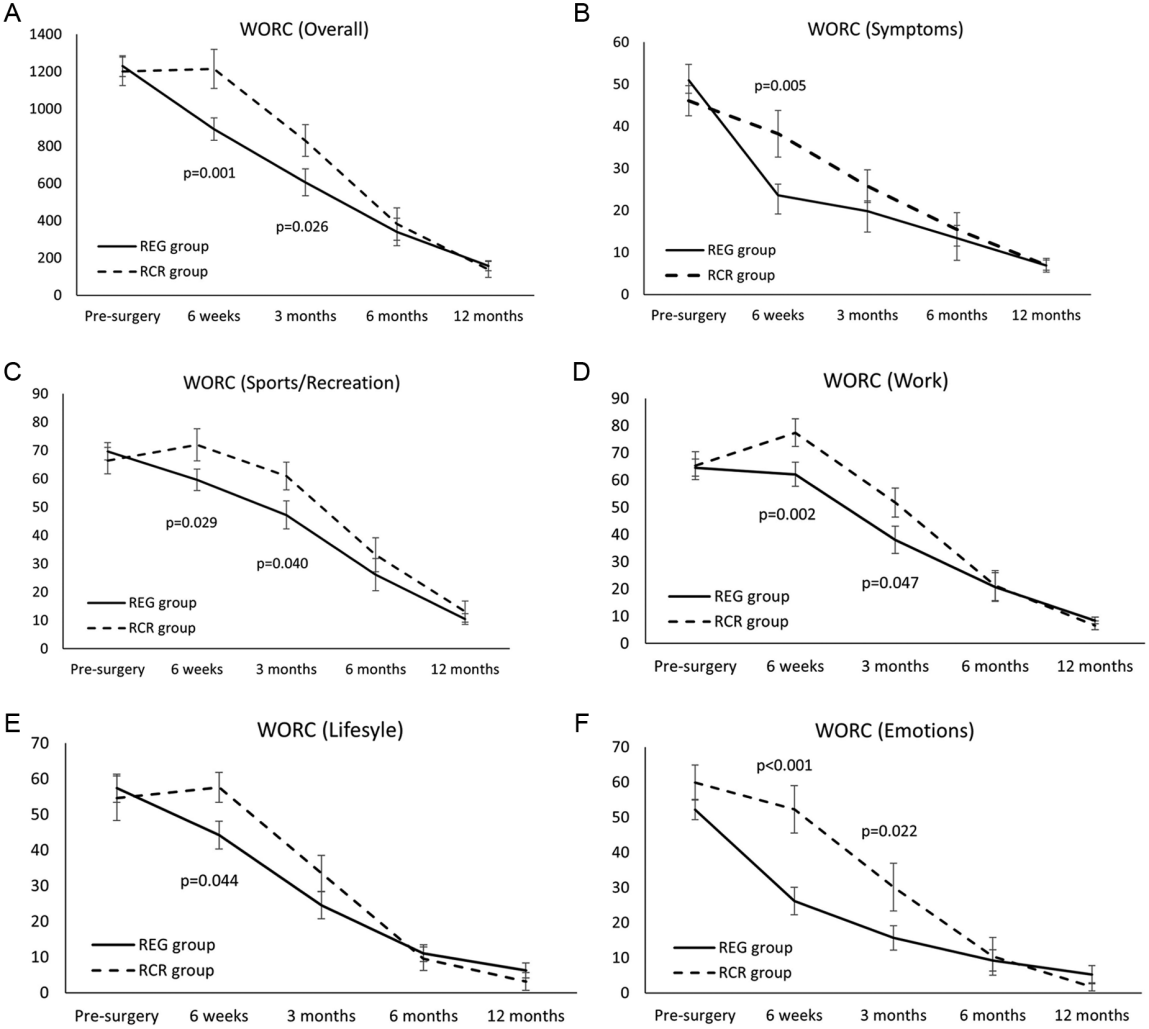
(A) Overall Western Ontario Rotator Cuff Index (WORC), as well as the individual WORC subscales including (B) Symptoms, (C) Sport and Recreation, (D) Work, (E) Lifestyle, and (F) Emotions, for patients randomized to the 2 groups: standard rotator cuff repair (RCR group) or augmentation of the rotator cuff tear with the bioinductive collagen implant (REG group).

The means and standard deviations of all PROMs are shown in Table 2. All PROMs (with the exception of the VR-12 MCS) significantly improved over time. A significant group effect was observed for the overall WORC (*P* = .045) as well as the Emotions subscale of the WORC (*P* = .023), whereas a significant group interaction effect was observed for the ASES, SANE, the overall WORC, and all WORC subscales (Table 2). Post hoc independent *t* tests for significant ANOVA effects demonstrated significantly better PROMs in the REG group for the total WORC at 6 weeks (*P* = .001) and 3 months (*P* = .026), which was the primary outcome measure. In addition, the WORC Symptoms subscale at 6 weeks (*P* = .005), the WORC Sport subscale at 6 weeks (*P* = .029) and 3 months (*P* = .040), the WORC Work subscale at 6 weeks (*P* = .002) and 3 months (*P* = .047), the WORC Lifestyle subscale at 6 weeks (*P* = .044), and the WORC Emotions subscale at 6 weeks (*P* < .001) and 3 months (*P* = .022) were superior in the REG versus RCR group. There were no group-based differences (*P* > .05) on any WORC subscale at 6 or 12 months after surgery. Furthermore, 19 (90.5%) REG patients and 13 (65%) RCR patients improved by more than the reported MID for the overall WORC at the primary 3-month endpoint. Figure 2 shows a graphical representation of the total WORC, as well as the individual subscales for both groups over the 12-month period.

**Table 2:** Patient-Reported Outcome Measures Over the Pre- and Postoperative Timeline for Patients Randomized to the 2 Groups*^*a*^*

	WORC Overall	WORC Symptoms	WORC Sport	WORC Work	WORC Lifestyle	WORC Emotions	ASES	SANE	VR-12 PCS	VR-12 MCS
Preoperative										
REG group	1229.2 ± 260.9	51.0 ± 17.6	69.7 ± 14.3	64.6 ± 14.6	57.4 ± 18.4	52.2 ± 13.6	51.5 ± 16.0	46.1 ± 20.3	41.8 ± 9.4	41.8 ± 9.6
RCR group	1201.5 ± 341.0	46.1 ± 16.1	66.4 ± 20.8	65.3 ± 23.2	54.6 ± 27.9	59.9 ± 22.3	57.3 ± 17.0	46.4 ± 17.5	41.7 ± 8.3	42.5 ± 6.4
6 wk postoperative										
REG group	890.7 ± 281.4	23.6 ± 12.6	59.6 ± 17.7	62.2 ± 20.9	45.9 ± 24.5	26.2 ± 18.4	68.2 ± 8.4	54.5 ± 16.9	43.0 ± 8.6	44.7 ± 7.8
RCR group	1250.6 ± 446.9	39.8 ± 24.2	72.8 ± 25.5	79.3 ± 21.4	59.8 ± 30.3	54.7 ± 28.8	50.2 ± 18.3	36.7 ± 22.4	39.1 ± 10.0	43.6 ± 6.3
3 mo postoperative										
REG group	605.4 ± 338.6	19.8 ± 11.3	47.2 ± 23.0	38.0 ± 23.5	24.6 ± 17.9	15.7 ± 16.4	73.7 ± 16.8	73.5 ± 16.4	50.7 ± 7.8	41.9 ± 6.3
RCR group	841.0 ± 386.7	26.0 ± 18.0	61.5 ± 22.3	52.4 ± 24.2	33.9 ± 23.0	31.4 ± 30.7	74.2 ± 11.5	65.0 ± 18.9	46.8 ± 9.9	43.0 ± 5.9
6 mo postoperative										
REG group	339.9 ± 346.9	13.4 ± 14.3	26.2 ± 26.4	20.7 ± 24.7	11.1 ± 11.1	9.2 ± 14.3	89.0 ± 8.8	82.1 ± 13.3	54.3 ± 8.4	41.3 ± 5.9
RCR group	381.9 ± 388.6	15.5 ± 17.7	33.2 ± 26.9	21.3 ± 24.5	9.6 ± 14.9	10.9 ± 24.4	90.6 ± 9.8	82.1 ± 17.7	55.8 ± 8.6	42.5 ± 4.8
12 mo postoperative										
REG group	162.7 ± 110.1	6.9 ± 5.9	10.5 ± 8.9	8.5 ± 5.5	7.0 ± 5.1	5.7 ± 5.7	92.7 ± 5.9	89.0 ± 9.5	55.6 ± 6.7	42.0 ± 5.3
RCR group	139.1 ± 149.4	7.0 ± 7.3	13.1 ± 16.9	6.7 ± 7.1	3.2 ± 6.8	1.8 ± 3.1	95.8 ± 6.2	92.8 ± 8.2	58.0 ± 5.5	42.1 ± 4.3
ANOVA results, *P*										
Time effect	<.001	<.001	<.001	<.001	<.001	<.001	<.001	<.001	<.001	.261
Group effect	.045	.180	.073	.080	.384	.023	.383	.144	.515	.519
Interaction effect	.023	.004	.036	.002	.002	<.001	<.001	.005	.050	.742

a Scores are expressed as mean ± SD. ANOVA, analysis of variance; ASES, American Shoulder and Elbow Surgeons Score; MCS, Mental Component Score; PCS, Physical Component Score; RCR, rotator cuff repair; REG, augmentation of the rotator cuff tear with the bioinductive collagen implant; SANE, Single Assessment Numeric Evaluation; VR-12, Veterans Rand 12-Item Health Survey; WORC, Western Ontario Rotator Cuff Index.

The ASES at 6 weeks (*P* < .001) and the SANE at 6 weeks (*P* = .003) were superior in the REG group, although there were no differences (*P* > .05) in these PROMs between groups at 3, 6, and 12 months. The pre- and postoperative means and standard deviations of the overall Constant score, as well as the individual Constant score components, are shown in Table 3. Although the overall Constant score, along with all individual Constant subscales, significantly improved (*P* < .001) over time, no group differences (*P* > .05) were observed (Table 3).

**Table 3:** Pre- and Postoperative Constant Subscale Scores and Total Scores for Patients Randomized to the 2 Groups*^*a*^*

	Pain	ADLs	ROM	Strength	Total Constant
Preoperative					
REG group	5.2 ± 1.9	8.6 ± 2.7	29.1 ± 6.6	8.1 ± 4.5	51.1 ± 10.9
RCR group	7.0 ± 3.3	10.0 ± 3.9	31.8 ± 6.3	8.7 ± 6.3	57.5 ± 14.3
6 mo postoperative					
REG group	12.9 ± 3.0	18.4 ± 2.5	35.8 ± 5.5	11.8 ± 5.0	78.3 ± 12.6
RCR group	13.3 ± 2.9	17.5 ± 2.6	36.2 ± 5.1	12.7 ± 6.9	79.1 ± 12.2
12 mo postoperative					
REG group	13.3 ± 2.4	19.3 ± 1.2	38.9 ± 1.7	14.7 ± 6.2	85.7 ± 13.9
RCR group	14.0 ± 3.0	19.2 ± 1.6	39.0 ± 1.6	16.2 ± 7.1	87.9 ± 13.3
ANOVA results, *P*					
Time effect	<.001	<.001	<.001	<.001	<.001
Group effect	.118	.840	.415	.533	.307
Interaction effect	.560	.293	.516	.443	.664

a Scores are expressed as mean ± SD. ADL, activities of daily living; ANOVA, analysis of variance; RCR, rotator cuff repair; REG, augmentation of the rotator cuff tear with the bioinductive collagen implant; ROM, range of motion.

The postoperative recovery timeline of specific functional activities as previously reported^16^ and collected throughout the current study is shown in Table 4. A significantly lower mean time to return to a number of events was observed in the REG group, including permanently removing the sling (*P* < .001), driving a car (*P* < .001), returning to office-based work (*P* = .009), and undertaking light (*P* = .007), moderate (*P* < .001), and high (*P* = .026) intensity household chores (Table 4).

**Table 4:** Postoperative Recovery Timeline (Weeks) of Relevant Variables and Activities Collected Throughout the Study, for Patients Randomized to the 2 Groups*^*a*^*

Activity	REG Group	RCR Group	*P*
Permanent sling removal	2.3 ± 1.6 (1-7)	5.3 ± 1.0 (2-6)	<.001
Driving a car	2.5 ± 1.9 (0.5-7)	5.1 ± 1.9 (1-8)	<.001
Office/desk work	1.7 ± 1.3 (0.5-5)	3.6 ± 2.9 (0.5-12)	.009
Light-intensity household chores	2.3 ± 1.6 (0.5-8)	4.2 ± 2.4 (2-12)	.007
Moderate-intensity household chores	3.8 ± 2.5 (0.5-12)	8.4 ± 5.0 (4-20)	<.001
High-intensity household chores	9.2 ± 7.3 (2-26)	15.0 ± 9.3 (6-32)	.026
Recreational/sport activities (speed & force)	19.7 ± 5.7 (8-26)	22.7 ± 7.1 (16-40)	.275
Recreational/sport activities (repetition)	19.2 ± 7.1 (12-32)	19.7 ± 2.7 (16-24)	.814

a Data are expressed as mean ± SD (range). RCR, rotator cuff repair; REG, augmentation of the rotator cuff tear with the bioinductive collagen implant.

### MRI-Based Scores

Preoperative MRI evaluation was performed to identify the configuration of the high-grade partial-thickness rotator cuff tear. The distributions of articular-sided (REG = 9; RCR = 8), bursal-sided (REG = 3; RCR = 3), and interstitial (REG = 9; RCR = 9) tears were similar across the 2 groups (Table 1).

Postoperatively, no difference was seen in MRI-based outcomes using the Sugaya classification system at 6-month (*P* = .201) or 12-month (*P* = .454) timepoints between the 2 groups (Table 5). At 6 months after surgery, 12 (57%) and 15 (75%) patients in the REG and RCR groups, respectively, were allocated Sugaya grade 1. At 12 months after surgery, 13 (62%) and 13 (65%) of the REG and RCR groups, respectively, were graded Sugaya 1 (Table 4). At 12 months after surgery, 19 (90%) and 17 (85%) REG and RCR patients, respectively, were graded Sugaya grade 1 or 2. No full-thickness supraspinatus tears (Sugaya grade 4 or 5) were noted in either treatment group.

**Table 5:** Magnetic Resonance Imaging–Based Sugaya Grading for 6- and 12-Month Images in Patients Randomized to the 2 Groups*^*a*^*

Timepoint	Outcome Measure	REG Group	RCR Group
6 months	Sugaya grading	1 (1-3)	1 (1-3)
	Grade 1	12	15
	Grade 2	5	3
	Grade 3	4	2
	Grade 4	0	0
	Grade 5	0	0
12 months	Sugaya grading	1 (1-3)	1 (1-3)
	Grade 1	13	13
	Grade 2	6	4
	Grade 3	2	3
	Grade 4	0	0
	Grade 5	0	0

a Shown is the median Sugaya grading (range), along with the number of patients graded within each of the Sugaya classifications (grades 1-5). RCR, rotator cuff repair; REG, augmentation of the rotator cuff tear with the bioinductive collagen implant.

### Complications, Adverse Events, Reinjuries, and/or Failures

Throughout the 12-month postoperative period, 1 patient in the REG group reported a fall when walking his dog at 6 weeks after surgery, which resulted in some immediate shoulder discomfort that had fully resolved within 4 weeks. A second patient in the RCR group reported a slip from a forklift at 4 months after surgery, which resulted in some immediate shoulder discomfort that had fully resolved within 6 weeks. One further patient in the REG group required a glenohumeral corticosteroid injection at 3 months for postoperative capsulitis.

## Discussion

The most important findings of the current study were that augmentation of symptomatic, high-grade, partial-thickness rotator cuff tears with a bioinductive collagen implant, compared with standard arthroscopic rotator cuff repair, resulted in superior 6-week and 3-month WORC scores, alongside a superior early improvement in other PROMs and a faster return to various work, ADLs, and recreational activities. Furthermore, MRI-based integrity of the treated rotator cuff tear demonstrated no differences at 12 months between the surgical treatments.

In an early study investigating the treatment of partial-thickness rotator cuff tears using a bioinductive collagen implant, Bokor et al^2^ reported significant improvements in postoperative ASES and Constant scores. In a later multicenter study of 33 patients, Schlegel et al^21^ reported significant improvements in the ASES and Constant scores at 2 years after surgery and with high rates of MRI-based cuff healing. A nonrandomized study published by Yeazell et al^30^ compared outcomes in patients undergoing bioinductive collagen implant treatment versus a matched cohort that underwent debridement, tear completion, and arthroscopic repair for high-grade partial-thickness rotator cuff tears. The authors reported that patients in the bioinductive collagen implant group had more stiffness at 12 weeks after surgery with a higher rate of second surgery compared with the standard arthroscopic repair group. In the current randomized controlled multicenter study, no patient in either treatment group required secondary surgery. Furthermore, significant improvements were observed in REG versus RCR group in the 3-month WORC score and WORC subscales, with 90.5% of the REG group, compared with 65% of the RCR group, improving by more than the MID for the overall WORC at 3 months after surgery.

Randomized controlled trials have been undertaken to evaluate the use of bioinductive collagen implants to augment full-thickness rotator cuff tears.^4,19^ To our knowledge, the current study is the first randomized controlled prospective trial to directly compare the treatment outcomes for high-grade partial-thickness rotator cuff tears using a bioinductive collagen implant versus standard arthroscopic repair. McIntyre et al^16^ reported 6-month ASES scores from the REBUILD multicenter registry and stated that patients treated with the bioinductive collagen implant for partial-thickness rotator cuff tears compared favorably to historical published studies using transtendon repair or takedown repair. Bushnell et al^3^ later reported a comparative study using the REBUILD multicenter registry data, comparing 241 patients undergoing isolated collagen implant augmentation versus 31 patients who underwent takedown repair and supplemental collagen implant augmentation. Bushnell et al specifically reported on early functional recovery with the intermediate- and high-grade rotator cuff tears, reporting that the takedown repair group (supplemented with collagen implant augmentation) had inferior PROMs at 2 and 6 weeks compared with the isolated collagen implant augmentation group. However, there were no differences between treatment groups in ASES and WORC scores at 3 and 12 months. Although the investigators concluded that isolated bioinductive collagen implant augmentation for partial-thickness rotator cuff tears offered improved clinical outcomes at a very early stage, they also indicated that takedown repair with additional collagen implant augmentation is not a conventional surgical approach for partial-thickness rotator cuff tears and was not an optimal comparator.

The current randomized study investigating bioinductive collagen implantation in the treatment of high-grade partial-thickness rotator cuff tears used standard arthroscopic cuff repair as the comparator and demonstrated superiority in the 3-month postoperative WORC score, the primary study outcome measure, and in support of the primary hypothesis, with equivalent rates of MRI-based tendon healing at 12 months after surgery. Another important strength of the current study was evaluation of the relative timing of recovery for specific daily, work, and recreational activities. McIntyre et al^16^ reported outcomes on some of these tasks after bioinductive collagen implant treatment including time using the sling (mean 10.6 days), time to drive a motor vehicle (mean 14.6 days), and time to return to sedentary (mean 9.4 days) and physical (mean 72.9 days) work. Bushnell et al^3^ later reported on these tasks, again using data from the REBUILD registry, including time to remove the sling (mean 19.1 days), return to driving (mean 17.1 days), and return to work (mean 33.3 days). These findings were similar to our study. However, it was demonstrated in the current randomized trial that REG versus RCR patients reported significantly less time in the sling (mean 2.3 vs 5.3 weeks), as well as a faster time to return to driving (mean 2.5 vs 5.1 weeks), time to return to office-based work (mean 1.7 vs 3.6 weeks), and time to undertake light (mean 2.3 vs 4.2 weeks), moderate (mean 3.8 vs 8.4 weeks), and high (mean 9.2 vs 15.0 weeks) intensity household chores. We acknowledge that the difference between the 2 surgical procedures and associated guidance on rehabilitation may have introduced some inherent bias with respect to the timing of return to some activities. Although direction was provided regarding use of the sling and the introduction of active ROM and strengthening exercises, in reality the progression toward home-based and work activities was at the individual patient's discretion and perceived readiness to proceed. Regardless, the current randomized trial provides valuable information that can be provided to patients regarding safe and realistic expectations of recovery and estimated timeframes to return to various work and recreational activities.

Importantly, the earlier return to function in the bioinductive collagen implant group did not come at the expense of inferior MRI-based outcomes. Preoperatively, all patients had high-grade (≥50%) partial-thickness supraspinatus tears (equivalent to Sugaya grade 3 tears), with the distribution of tear configurations comparable between the REG and RCR groups. At 6 months after surgery, only 4 (19%) REG patients (2 articular, 1 bursal, and 1 interstitial tear) remained with a Sugaya grade 3. At 12 months, the 2 articular tears remained grade 3, with the interstitial and bursal tears improving 1 grade. In the RCR group, only 2 (10%) patients (1 articular and 1 bursal) remained with a Sugaya grade 3 at 6 months after surgery, although this had increased to 3 patients at 12 months. Importantly, at 12 months there was no difference between groups in MRI-based rotator cuff healing, with no full-thickness rotator cuff tears and 90% of the REG group and 85% of the RCR group improving by at least 1 Sugaya grade. This is consistent with the study by Schlegel et al,^21^ who reported substantial new tissue infill on MRI scans in 84% of high-grade rotator cuff tears and 91% of intermediate-grade tears in patients undergoing bioinductive collagen implant augmentation. Only 1 patient progressed from a high-grade partial-thickness rotator cuff tear to a full-thickness tear. In the current study, the number of radiological failures (Sugaya grade ≥3) observed was too small to ascertain whether supraspinatus healing with bioinductive collagen implant augmentation is more reliable with bursal, interstitial, or articular tear patterns. Furthermore, the mechanism of tendon healing and symptom resolution with bioinductive collagen implant augmentation is likely to differ from rotator cuff takedown and repair. Although healing of the tendon bone interface of articular sided tears in particular may be incomplete with bursal implant placement, the thicker tendon anterior and posterior to the residual partial-thickness rotator cuff tear may reduce peak intratendinous strain and associated symptoms.^18,20^

An important confounding factor in the current study that may have contributed to superiority in early functional recovery of the REG group was the difference in sling immobilization time and rehabilitation protocols. Patients in the REG group were advised to wear a sling full-time for 7 days as per the postoperative protocol published in previous studies.^3,16^ For patients in the RCR group, sling use for 5 weeks was advised, consistent with traditional rotator cuff repair rehabilitation protocols.^3,17,23^ Notably, in the comparative study undertaken by Bushnell et al,^3^ the cohort that underwent takedown repair with bioinductive collagen implant augmentation wore a sling for a mean 34 days after surgery. However, there is now emerging evidence to suggest that sling time after standard rotator cuff repair can be safely reduced, and this may lead to improved early functional recovery.^10^ Tirefort et al^25^ undertook a randomized trial on the use of sling immobilization after cuff repair for small and medium-sized rotator cuff tears. Those investigators reported that although no sling use (vs the control group with 4 weeks of sling use) resulted in superior early active ROM, as well as a lower visual analog pain score and better SANE score at 6 months, there was no difference in ASES and SANE scores at 3 months after surgery. In the current study, the REG group wore a sling for a mean of 2.3 weeks and the RCR group wore a sling for a mean 5.3 weeks; PROMs were superior in the REG group at 3 months after surgery, suggesting that the difference in sling time was not critical to 3-month difference in functional outcome. Nevertheless, it is acknowledged that the less restrictive rehabilitation protocol in the REG group may have contributed to the superior PROMs at 3 months after surgery. Further research may look to use an identical sling and rehabilitation protocol for patients with partial-thickness rotator cuff tears treated with either bioinductive collagen implant augmentation or standard cuff repair.

In the current study, complications in both treatment groups were low. There were no infections or secondary surgeries. In the REG group, 1 patient underwent a glenohumeral cortisone injection at 3 months after surgery for capsulitis, which subsequently resolved. McIntyre et al^16^ reported that 32% of patients needed cortisone injections after bioinductive collagen implant augmentation, although the use of steroid injections was surgeon and center dependent. The current study used strict inclusion criteria, and caution was taken to exclude concomitant adhesive capsulitis before patients were enrolled. Enrolled patients had shoulder symptoms for a mean of 36 months, further reducing the possibility of frozen shoulder being a concomitant diagnosis. No adverse reactions to the bioinductive collagen implant were observed, and a recent systematic review suggested that these are rare.^26^ Likewise, the current study did not recognize any florid subacromial bursitis or implant dislodgement, with the aforementioned systematic review reporting an overall revision surgery rate of 1.0% to 4.0% after implant placement for partial-thickness rotator cuff tears.

### Limitations

The current study is not without limitations. First, the confounding effect of different rehabilitation protocols between treatment groups has been discussed above. Second, the small sample size in this randomized prospective trial is acknowledged. The study was powered based on a large anticipated between-group effect in the 3-month WORC score, the primary study outcome measure, for which an initial sample size calculation was performed. It is acknowledged that the current study was not powered for other reported outcomes, including the Constant score or MRI-based outcomes. Our primary outcome measure, the 3-month overall WORC score, was a clinical continuous variable, rather than a dichotomous variable, so a study fragility score could not be calculated. Nonetheless, no patients were lost to follow-up or withdrawn from the study. Third, the inclusion and exclusion criteria in the current study were strict, which may limit the generalizability to a more heterogeneous cohort that may include smokers and workers’ compensation patients who are reported as doing less well after rotator cuff repair surgery.^3^ Fourth, this randomized trial was not double-blinded; both the patients and independent research study assessors were aware of the treatment allocation of participants. The musculoskeletal radiologist evaluating the postoperative MRI scans was not informed of the treatment allocation, but the presence of suture anchors on MRI would have indicated the RCR group allocation. The lack of double-blinding relegates this randomized controlled surgical trial to a level 2 study. Fifth, 4 orthopaedic surgeons were included in the study. Although this may be seen as a limitation, the generalizability and multicenter nature are strengths.

## Conclusion

In the treatment of high-grade partial-thickness rotator cuff tears, bioinductive collagen implant augmentation, compared with standard arthroscopic rotator cuff repair, improved early symptoms and functional recovery, with equivalent rates of tendon healing on MRI at 12 months after surgery.

## References

[1] Bauer S Wang A Butler R , et al. Reliability of a 3 T MRI protocol for objective grading of supraspinatus tendonosis and partial thickness tears. J Orthop Surg Res. 2014;9:128. 25519001 10.1186/s13018-014-0128-x PMC4278262

[2] Bokor DJ Sonnabend D Deady L , et al. Evidence of healing of partial-thickness rotator cuff tears following arthroscopic augmentation with a collagen implant: a 2-year MRI follow-up. Muscles Ligaments Tendons J. 2016;6(1):16-25. 27331028 10.11138/mltj/2016.6.1.016 PMC4915456

[3] Bushnell BD Bishai SK Krupp RJ , et al. Treatment of partial-thickness rotator cuff tears with a resorbable bioinductive bovine collagen implant: 1-year results from a prospective multicenter registry. Orthop J Sports Med. 2021;9(8):23259671211027850. 34409115 10.1177/23259671211027850 PMC8366148

[4] Camacho Chacon JA Roda Rojo V Martin Martinez A , et al. An isolated bioinductive repair vs sutured repair for full-thickness rotator cuff tears: 2-year results of a double blinded, randomized controlled trial. J Shoulder Elbow Surg. 2024;33(9):1894-1904. 38734130 10.1016/j.jse.2024.03.043

[5] Castricini R La Camera F De Gori M , et al. Functional outcomes and repair integrity after arthroscopic repair of partial articular supraspinatus tendon avulsion. Arch Orthop Trauma Surg. 2019;139(3):369-375. 30269221 10.1007/s00402-018-3044-4

[6] Constant CR Gerber C Emery RJ Sojbjerg JO Gohlke F Boileau P. A review of the Constant score: modifications and guidelines for its use. J Shoulder Elbow Surg. 2008;17(2):355-361. 18218327 10.1016/j.jse.2007.06.022

[7] Constant CR Murley AH . A clinical method of functional assessment of the shoulder. Clin Orthop Relat Res. 1987(214):160-164. 3791738

[8] Cucchi D Menon A Maggi S , et al. Treatment of partial rotator cuff lesions is associated with a higher frequency of post-operative shoulder stiffness: a prospective investigation on the role of surgery-related risk factors for this complication. Arch Orthop Trauma Surg. 2022;142(11):3379-3387. 34905067 10.1007/s00402-021-04285-1 PMC9522663

[9] Gagnier JJ Robbins C Bedi A Carpenter JE Miller BS. Establishing minimally important differences for the American Shoulder and Elbow Surgeons score and the Western Ontario Rotator Cuff Index in patients with full-thickness rotator cuff tears. J Shoulder Elbow Surg. 2018;27(5):e160-e166. 10.1016/j.jse.2017.10.042 29307675

[10] Houck DA Kraeutler MJ Schuette HB McCarty EC Bravman JT. Early versus delayed motion after rotator cuff repair: a systematic review of overlapping meta-analyses. Am J Sports Med. 2017;45(12):2911-2915. 28288280 10.1177/0363546517692543

[11] Jordan RW Bentick K Saithna A. Transtendinous repair of partial articular sided supraspinatus tears is associated with higher rates of stiffness and significantly inferior early functional scores than tear completion and repair: a systematic review. Orthop Traumatol Surg Res. 2018;104(6):829-837. 30036723 10.1016/j.otsr.2018.06.007

[12] Kamath G Galatz LM Keener JD Teefey S Middleton W Yamaguchi K. Tendon integrity and functional outcome after arthroscopic repair of high-grade partial-thickness supraspinatus tears. J Bone Joint Surg Am. 2009;91(5):1055-1062. 19411453 10.2106/JBJS.G.00118

[13] Keener JD Galatz LM Teefey SA , et al. A prospective evaluation of survivorship of asymptomatic degenerative rotator cuff tears. J Bone Joint Surg Am. 2015;97(2):89-98. 25609434 10.2106/JBJS.N.00099 PMC4296477

[14] Kirkley A Alvarez C Griffin S. The development and evaluation of a disease-specific quality-of-life questionnaire for disorders of the rotator cuff: the Western Ontario Rotator Cuff Index. Clin J Sport Med. 2003;13(2):84-92. 12629425 10.1097/00042752-200303000-00004

[15] Lo IK Denkers MR More KD Nelson AA Thornton GM Boorman RS. Partial-thickness rotator cuff tears: clinical and imaging outcomes and prognostic factors of successful nonoperative treatment. Open Access J Sports Med. 2018;9:191-197. 30271226 10.2147/OAJSM.S153236 PMC6149897

[16] McIntyre LF Bishai SK Brown PB III Bushnell BD Trenhaile SW. Patient-reported outcomes after use of a bioabsorbable collagen implant to treat partial and full-thickness rotator cuff tears. Arthroscopy. 2019;35(8):2262-2271. 31350082 10.1016/j.arthro.2019.02.019

[17] Peters KS McCallum S Briggs L Murrell GA. A comparison of outcomes after arthroscopic repair of partial versus small or medium-sized full-thickness rotator cuff tears. J Bone Joint Surg Am. 2012;94(12):1078-1085. 22717826 10.2106/JBJS.J.00519

[18] Reilly P Amis AA Wallace AL Emery RJ. Supraspinatus tears: propagation and strain alteration. J Shoulder Elbow Surg. 2003;12(2):134-138. 12700564 10.1067/mse.2003.7

[19] Ruiz Iban MA Garcia Navlet M Moros Marco S , et al. Augmentation of a posterosuperior cuff repair with a bovine bioinductive collagen implant shows a lower retear rate but similar outcomes compared with no augmentation: 2-year results of a randomized controlled trial. Arthroscopy. 2025;41(10):3869-3879. 40209829 10.1016/j.arthro.2025.03.057

[20] Sano H Wakabayashi I Itoi E. Stress distribution in the supraspinatus tendon with partial-thickness tears: an analysis using two-dimensional finite element model. J Shoulder Elbow Surg. 2006;15(1):100-105. 16414477 10.1016/j.jse.2005.04.003

[21] Schlegel TF Abrams JS Angelo RL Getelman MH Ho CP Bushnell BD. Isolated bioinductive repair of partial-thickness rotator cuff tears using a resorbable bovine collagen implant: two-year radiologic and clinical outcomes from a prospective multicenter study. J Shoulder Elbow Surg. 2021;30(8):1938-1948. 33220413 10.1016/j.jse.2020.10.022

[22] Sher JS Uribe JW Posada A Murphy BJ Zlatkin MB. Abnormal findings on magnetic resonance images of asymptomatic shoulders. J Bone Joint Surg Am. 1995;77(1):10-15. 7822341 10.2106/00004623-199501000-00002

[23] Shin SJ. A comparison of 2 repair techniques for partial-thickness articular-sided rotator cuff tears. Arthroscopy. 2012;28(1):25-33. 22000411 10.1016/j.arthro.2011.07.005

[24] Sugaya H Maeda K Matsuki K Moriishi J. Repair integrity and functional outcome after arthroscopic double-row rotator cuff repair. A prospective outcome study. J Bone Joint Surg Am. 2007;89(5):953-960. 17473131 10.2106/JBJS.F.00512

[25] Tirefort J Schwitzguebel AJ Collin P Nowak A Plomb-Holmes C Ladermann A. Postoperative mobilization after superior rotator cuff repair: sling versus no sling: a randomized prospective study. J Bone Joint Surg Am. 2019;101(6):494-503. 30893230 10.2106/JBJS.18.00773

[26] Warren JR Domingo-Johnson ER Sorensen AA Cheng AL Latz KH Cil A. Bioinductive patch as an augmentation for rotator cuff repair, a systematic review and meta-analysis. J Shoulder Elbow Surg. 2024;33(11):2515-2529. 38942225 10.1016/j.jse.2024.05.002

[27] Waterman BR Newgren J Gowd AK , et al. Randomized trial of arthroscopic rotator cuff with or without acromioplasty: no difference in patient-reported outcomes at long-term follow-up. Arthroscopy. 2021;37(10):3072-3078. 33940126 10.1016/j.arthro.2021.04.041

[28] Woodmass JM Al Khatib L McRae S , et al. Arthroscopic rotator cuff repair with and without acromioplasty in the treatment of full-thickness rotator cuff tears: long-term outcomes of a multicenter, randomized controlled trial. J Bone Joint Surg Am. 2022;104(23):2101-2107. 36476738 10.2106/JBJS.22.00135

[29] Yang S Pang L Zhang C , et al. Lower reoperation rate and superior patient-reported outcome following arthroscopic rotator cuff repair with concomitant acromioplasty: an updated systematic review of randomized controlled trials. Arthroscopy. 2025;41(5):1618-1634. 38876445 10.1016/j.arthro.2024.05.026

[30] Yeazell S Lutz A Bohon H , et al. Increased stiffness and reoperation rate in partial rotator cuff repairs treated with a bovine patch: a propensity-matched trial. J Shoulder Elbow Surg. 2022;31(6S):S131-S135. 10.1016/j.jse.2022.02.003 35288296

[31] Zhou L Natarajan M Miller BS Gagnier JJ. Establishing minimal important differences for the VR-12 and SANE scores in patients following treatment of rotator cuff tears. Orthop J Sports Med. 2018;6(7):2325967118782159. 10.1177/2325967118782159 PMC6077909 30090834

